# Hydrogen Production by a *Chlamydomonas reinhardtii* Strain with Inducible Expression of Photosystem II

**DOI:** 10.3390/ijms18030647

**Published:** 2017-03-16

**Authors:** Khorcheska Batyrova, Patrick C. Hallenbeck

**Affiliations:** 1 Département de Microbiologie, Infectiologie et Immunologie, Université de Montréal, CP6128 Succursale Centre-ville, Montréal, Québec, QC H3C 3J7, Canada; horcheska@gmail.com; 2 Life Sciences Research Center, Department of Biology, United States Air Force Academy, Colorado Springs, CO 80840, USA

**Keywords:** biohydrogen, biophotolysis, *Chlamydomonas reidhardtii*, inducible promotor

## Abstract

*Chlamydomonas reinhardtii* cy6Nac2.49 is a genetically modified algal strain that activates photosynthesis in a cyclical manner, so that photosynthesis is not active constitutively in the presence of oxygen, but is turned on only in response to a metabolic trigger (anaerobiosis). Here, we further investigated hydrogen production by this strain comparing it with the parental wild-type strain under photoheterotrophic conditions in regular tris-acetate-phosphate (TAP) medium with a 10-h:14-h light/dark regime. Unlike the wild-type, whose level of H_2_ production remained low during illumination, H_2_ production in the mutant strain increased gradually with each subsequent light period, and by the final light period was significantly higher than the wild-type. The relatively low Photosystem II (PSII) activity of the mutant culture was shown by low fluorescence yield both in the dark (Fv/Fm) and in the light (δF/Fm’) periods. Measurement of oxygen evolution confirmed the low photosynthetic activity of the mutant cells, which gradually accumulated O_2_ to a lesser extent than the wild-type, thus allowing the mutant strain to maintain hydrogenase activity over a longer time period and to gradually accumulate H_2_ during periods of illumination. Therefore, controllable expression of PSII can be used to increase hydrogen production under nutrient replete conditions, thus avoiding many of the limitations associated with nutrient deprivation approaches sometimes used to promote hydrogen production.

## 1. Introduction

The ability of green algae to produce hydrogen in the light was discovered nearly 75 years ago by Gaffron and Rubin, 1942 [1]. Since then, a variety of phototrophic microorganisms, including numerous species of *Chlamydomonas*, have been shown to produce H_2_. *Chlamydomonas reinhardtii* (*C. reinhardtii*) uses light for input energy and CO_2_ as a feedstock to oxidize water to produce organic carbon compounds and nicotinamide adenine dinucleotide phosphate (NADPH) for energy. This creates a paradox for the production of biohydrogen from *C. reinhardtii* since photosynthesis provides the building blocks for hydrogen gas production (protons and electrons) but also generates oxygen, an inhibitor of hydrogen gas production. Oxygen both inactivates the [Fe–Fe] hydrogenase enzyme that catalyzes H_2_ production, and inhibits the transcription of the genes encoding the [Fe–Fe] hydrogenase enzyme [2]. Hence in nature, the biological production of hydrogen is limited to a short burst that occurs when dark-adapted cultures are exposed to light. During the dark period, photosynthesis does not occur and the residual dissolved oxygen present in the surrounding aqueous environment is consumed by mitochondrial respiration [3]. This leads to the transcription and translation of the hydrogenase enzyme, which when active, produces hydrogen for only a brief moment in response to light until oxygen is produced to inhibitory concentrations. Therefore, in order to produce biological hydrogen using *C. reinhardtii,* specific strategies are required that separate photosynthesis and hydrogen production in time and/or space. 

One approach to separate photosynthesis and hydrogen production in time involves the use of nutrient-deprivation. It has been shown that the absence of essential macronutrients and micronutrients in the culture environment, such as sulfur, nitrogen, phosphorus or magnesium leads to gradual inactivation of Photosystem II (PSII) [4,5,6,7]. Under these conditions, oxygen evolution ceases and residual oxygen is depleted through respiration. This process leads to anaerobiosis, which in turn induces the synthesis of hydrogenase with subsequent H_2_ production. Moreover, nutrient starvation leads to the accumulation of carbohydrates, important for sustained hydrogen production in the long term [8,9]. It also leads to the inhibition of the Calvin–Benson cycle, thereby removing a significant electron sink and thus favoring hydrogen production [10]. However, cells can only survive for a few days in nutrient-depleted medium and will eventually die. Additionally, it is generally thought that in order to obtain high levels of H_2_ production by green algae, light conversion efficiencies will need to be increased. However, the degradation of PSII under nutrient-deprivation leads to a decrease in light conversion efficiencies, especially under high, natural, light conditions. 

In the present study we wished to further examine the properties of a strain in which *psbD*, encoding the D2 protein of PSII, is expressed under anaerobic conditions [11,12]. In order to develop an anaerobically inducible system for *psbD*, the nucleus-encoded chloroplast Nac2 protein was used. This protein binds to the 74 nucleotide psbD 5′ UTR and is therefore necessary for processing and stable accumulation of psbD mRNA. In the cy6Nac2.49 construct, the Nac2 coding sequence is fused to the cytochrome c6 (Cyc6) promoter, whose expression is induced by anaerobiosis. Since the background of this strain is nac2-26 (a non-functional allele of nac2, which encodes a protein absolutely required for *psbD* messengaer RNA maturation), the only functional Nac2 present is that produced from the construct under anaerobic conditions. In this system PSII synthesis can be regulated in a reversible manner while maintaining all other photosynthetic subunits active in the thylakoid membrane. The advantage of this system is that anaerobiosis can be achieved using cultures grown in nutrient-replete medium. Under these conditions, the cells should in principle remain healthy. This approach thus differs from the classical method in which PSII is inactivated through nutrient depletion, a condition that leads to impairment of cell growth and eventually to cell death. Here, we demonstrate that PSII controllable expression system can improve H_2_ production in green algae without the application of nutrient deprivation, therefore avoiding limitations inherent in nutrient deprivation approaches.

## 2. Results

### 2.1. Cell Growth under Photoheterotrophic, Photomixotrophic and Autotrophic Conditions

**Figure 1:**
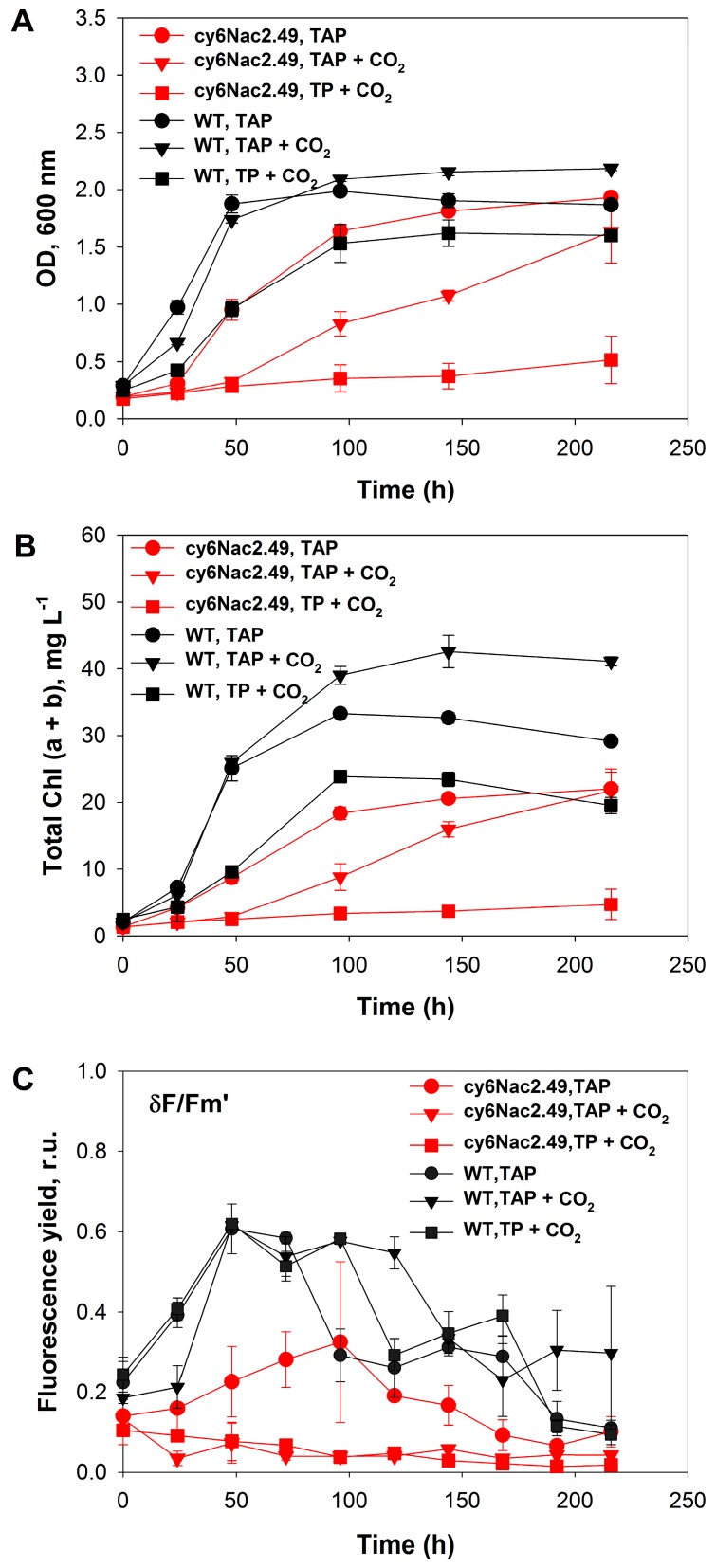
Growth and maximum photosynthetic yield during continuous illumination (10 W·m^−2^) of *C. reinhardtii* strain cy6Nac2.49 (red) and it is parental wild-type (black) under photoheterotrophic (TAP), photomixotrophic (TAP + CO_2_) and autotrophic (TP + CO_2_) conditions. (**A**) optical density (OD), (**B**) the total (a + b) chlorophyll (Chl) concentration and (**C**) the δFv/Fm’. TAP tris-acetate-phosphate medium; TP: tris-phosphate medium; δFv/Fm’: the maximum fluorescence value recorded during illumination; r.u.: relative units.

A series of experiments were conducted to compare the mutant strain’s physiology with that of the parental wild-type under photoheterotrophic, photomixotrophic and autotrophic conditions with a light intensity of 10 W·m^−2^ (48 μmol m^−2^·s^−1^). The use of regular tris-acetate-phosphate (TAP) medium provided photoheterotrophic conditions under which to compare the growth and chlorophyll content of the mutant and wild-type strains, the addition of CO_2_ to the head space (~40% final) under these conditions allowed the observation of growth under mixotrophic conditions, and the use of CO_2_ supplementation with tris-phosphate (TP) medium provided photoautotrophic conditions. Under photoheterotrophic conditions ( TAP medium), cultures of the mutant strain and the wild-type at longer time points (≥100 h) had similar cell concentrations based on OD_600_ levels (Figure 1A), however the mutant strain had a lower cellular chlorophyll (Chl) content than wild-type cells at all time points examined (Figure 1B). OD_600_ gives a good indication of biomass concentration since this wavelength is at a minimum in in vivo chlorophyll absorbance. Under photomixotrophic conditions, the mutant strain consumed acetate and gradually accumulated biomass, and after 200 h of incubation, had a similar cell concentration as it had under photoheterotrophic conditions. However, under photomixotrophic conditions the mutant strain had lower OD levels and cellular Chl content than wild-type cells at all time points examined (Figure 1A,B).

**Figure 2:**
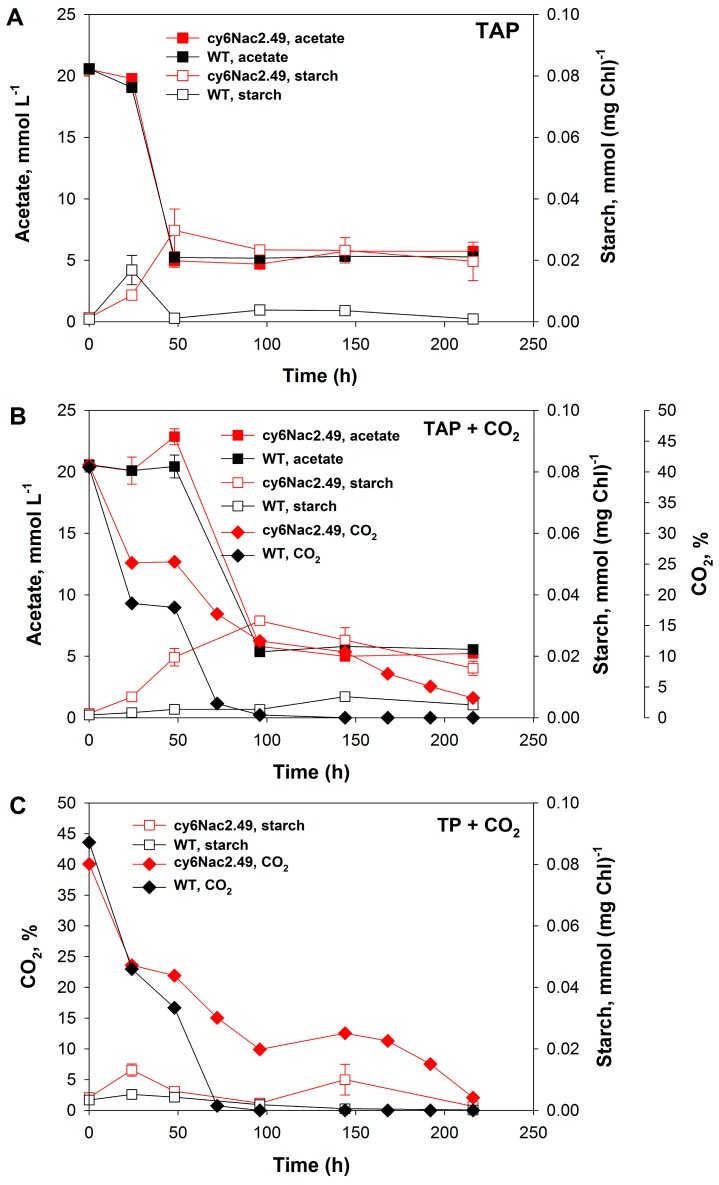
Changes in the amount of starch accumulated, the acetate content of the medium and amount of CO_2_ in the gas phase during the incubation of *C. reinhardtii* strain cy6Nac2.49 (red) and it is parental wild-type (black) under (**A**) photoheterotrophic (TAP); (**B**) photomixotrophic (TAP + CO_2_); and (**C**) autotrophic (TP + CO_2_) growth conditions under continuous illumination (10 W·m^−2^).

Significant differences in growth and CO_2_ consumption activities of the mutant and wild-type cells (Figure 1A,B and Figure 2B,C) were seen under photomixotrophic and photoautotrophic conditions with CO_2_ addition (to final concentration in the gas phase of ~40%). Sealed vials with a known initial concentration of carbon dioxide (40%) were used in order to accurately follow its consumption. No carbon dioxide additions were made during the experiment. Under photoautotrophic conditions, the wild-type strain showed rapid growth before entering stationary phase when the medium had become CO_2_ depleted (Figure 1A,B and Figure 2C), whereas with the mutant strain a delay in the transition from the exponential to the stationary growth phase and continuous CO_2_ consumption was observed under photomixotrophic and photoautotrophic conditions (Figure 1A,B and Figure 2B,C). Interestingly, the mutant strain appeared incapable of significant photoautotrophic growth under the conditions tested. In fact, addition of CO_2_ to TAP medium appeared to cause inhibition since both OD_600_ and the cellular chlorophyll content were lower when the mutant was grown photomixotrophically as opposed to photoheterotrophically.

The inability of the mutant to grow photosynthetically with CO_2_ is further highlighted by the very low maximum photosynthetic yields δFv/Fm’ determined during continuous illumination (10 W·m^−2^) under photoautotrophic and photomixotrophic growth conditions (Figure 1C). The low photosynthetic yields, measured as the maximum fluorescence value δFv/Fm’ recorded during illumination, indicates a low activity of PSII in these cells, expected to some degree for this strain with controllable expression of PSII. It would appear that the impaired PSII in the mutant reduces the photosynthetic production of NADPH and adenosine triphosphate (ATP), thereby restricting the amount of CO_2_ that can be reduced to glucose by the Calvin-Benson cycle. Likewise, a low but continuous CO_2_ consumption by the mutant strain was observed, very unlike the rapid CO_2_ uptake under these conditions by wild-type cultures. CO_2_ was completely consumed by the wild-type during ~50 h whereupon the culture went into growth arrest. On the other hand, cultures of the mutant strain were consuming CO_2_ for ~200 h and CO_2_ was not completely depleted at the end of experiment (Figure 1A,B and Figure 2B,C).

It is well known that acclimation of algal cells to nutrient-deprived conditions is accompanied by the accumulation of starch during the O_2_-production stage and its degradation during the H_2_ production stage [4,13,14]. However, here nutrient deprivation approaches were not applied. Therefore, significant starch accumulation by either the mutant or wild-type cultures did not occur, and newly assimilated carbon was apparently shifted towards biomass accumulation (Figure 2A–C). The kinetics of starch accumulation and degradation are included here to allow comparison with other work, but the absolute levels are much lower than what has previously been observed with nutrient deprived cultures, raising serious doubts as to the significance of starch metabolism in hydrogen production in these cultures.

### 2.2. Effects of Different Light Intensities under a Light/Dark Regime

**Figure 3:**
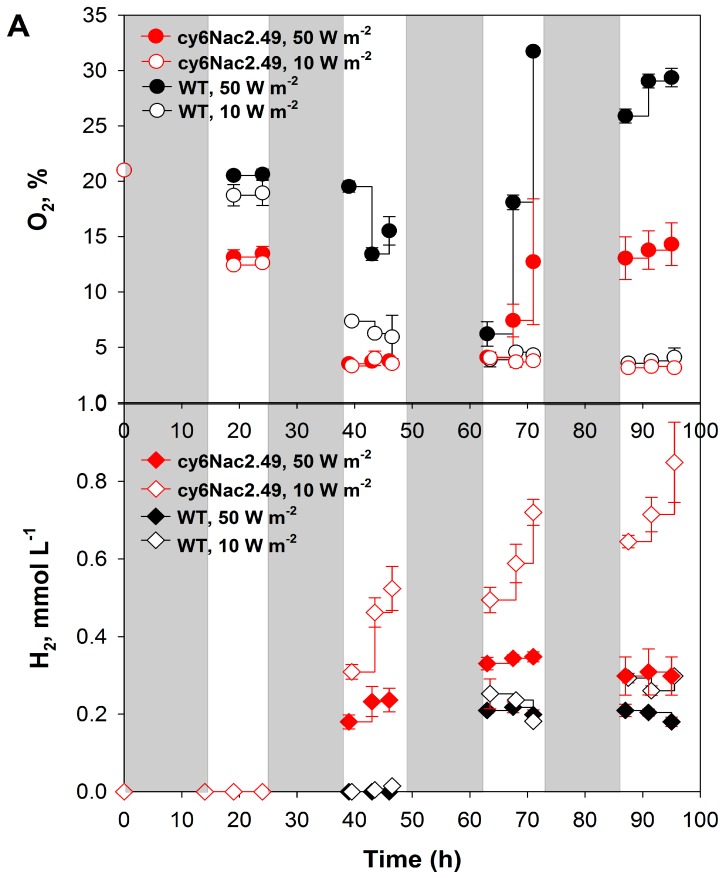

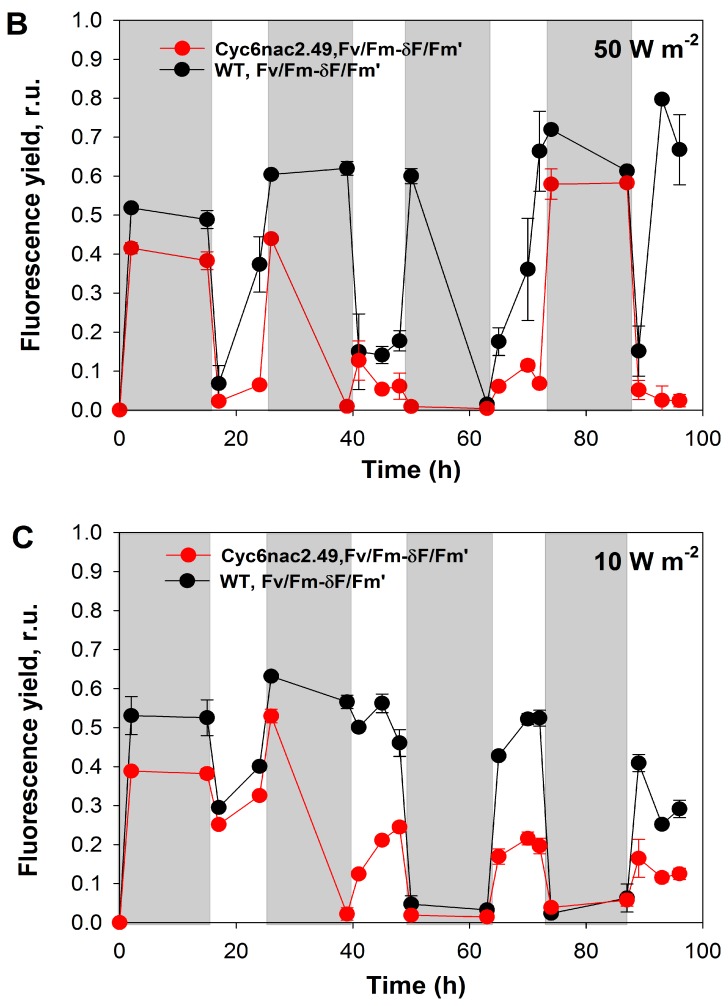
(**A**) H_2_ and O_2_ production of *C. reinhardtii* strain cy6Nac2.49 (red) and it is parental wild-type (black) under photoheterotrophic conditions on TAP medium under a light/dark regime at light intensities of 50 W·m^−2^ and 10 W·m^−2^. Cultures were left under an air atmosphere in sealed vials at the start of the experiment and were not sparged with argon during the experiment. The maximum photosynthetic yields were measured at different light intensities (**B**) 50 W·m^−2^ and (**C**) 10 W·m^−2^ respectively, where Fv/Fm represents the maximum fluorescence emission recorded in the dark periods, while δFv/Fm’ represents the maximum fluorescence value recorded during illumination. Shaded and not shaded sections represent dark (14 h) and light (10 h) periods respectively.

A series of experiments were performed to examine how different light intensities might affect the cy6Nac2.49 strain A 10-h:14-h light:dark cycle was used to more closely mimic natural conditions. As well, light dark periods have been suggested to increase photosynthetic efficiencies [15]. Cultures were incubated under an air atmosphere in sealed vials in TAP medium under two different light intensities, 10 and 50 W·m^−2^. H_2_ production was not observed in the first light period under either light intensity (10 and 50 W·m^−2^). Presumably this was due to the high initial concentration of O_2_ in the headspace (21%), too much for these relatively low density cultures to be able to completely respire during the first 14 h of dark incubation. In the second and subsequent light cycles, wild-type cultures showed the classic hydrogen burst when dark-adapted anaerobic cells were illuminated, but then cells quickly accumulate significant amounts of oxygen due to the reactivation of PSII, thereby inhibiting hydrogenase activity and H_2_ production. Thus, the overall oxygen production rate by the wild-type was higher than in cultures of the cy6Nac2.49 strain under both light intensities (Figure 3A). Measurement of the head space oxygen concentrations during subsequent light periods showed low, but appreciable levels of oxygen. Although this might seem paradoxical in face of hydrogen production by these strains, we attribute this to a lag in the reflection of the true liquid gas composition caused by liquid-gas mass transfer restrictions.

Unlike the wild-type, which did not produce appreciable levels of H_2_ during the second light period and only produced a low level in the following light period, H_2_ production by the cy6Nac2.49 strain continuously increased with each subsequent light period, producing significantly higher total amounts of H_2_ (Figure 3A). Remarkably, much higher H_2_ production in the mutant was observed under lower light intensity (10 W·m^−2^ (48 μmol·m^−2^·s^−1^) relative to 50 W·m^−2^ (240 μmol·m^−2^·s^−1^). As can be seen from Figure 3A in the last two light periods cultures of the cy6Nac2.49 strain were producing higher amounts of O_2_ under 50 W·m^−2^ then under 10 W·m^−2^ and therefore less H_2_ relative to production of H_2_ under 10 W·m^−2^.

Only low levels of photosynthetic yield, measured as the maximum fluorescence emission Fv/Fm, recorded in dark periods, and δFv/Fm’, the maximum fluorescence value recorded during illumination, were observed for the cy6Nac2.49 strain at both light intensities (Figure 3B,C). Although the initial values of the two cultures were roughly similar, there was a large divergence over repeated light:dark cycles reflecting the very different regulation of PSII and a divergence in PSII activity that grew with time. This no doubt reflects the relatively low activity and rapid turnover of PSII in cy6Nac2.49 cells. Therefore, it can be suggested that, due to the low photosynthetic activity, cy6Nac2.49 cells only gradually accumulated O_2_ to a lesser extent than the wild-type, enabling the cy6Nac2.49 strain to maintain hydrogenase activity longer and with gradual production and accumulation of H_2_ during the periods of illumination. On the other hand, the wild-type demonstrated the classical hydrogen burst within the first several minutes, with subsequent immediate inactivation of hydrogenase.

**Figure 4:**
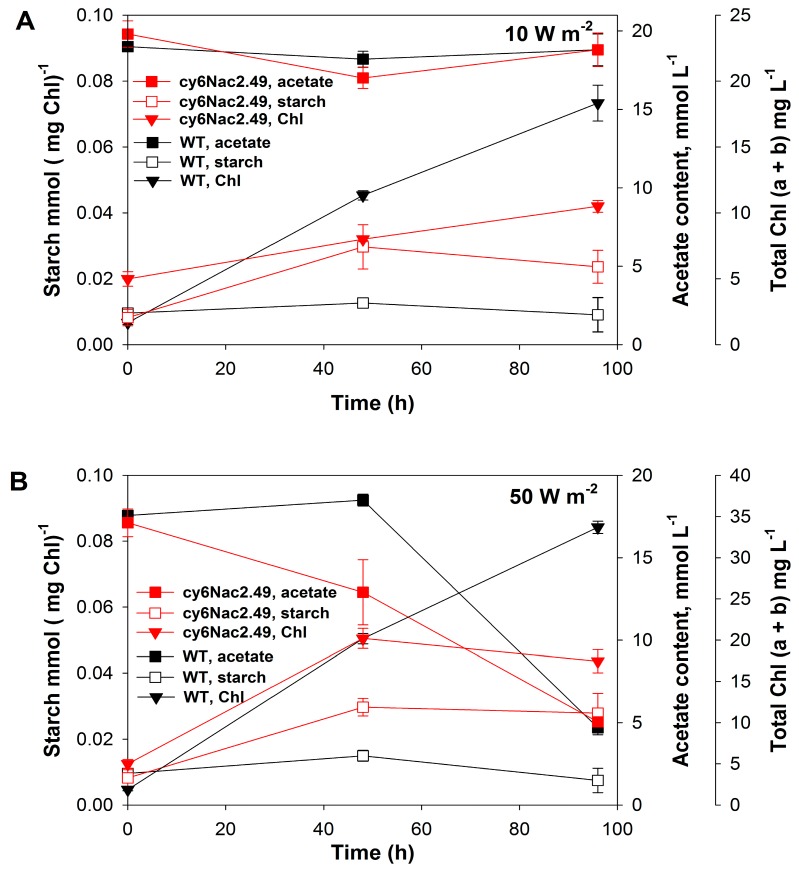
Changes in the total (a + b) chlorophyll (Chl) concentration, the amount of accumulated starch and the acetate content in the medium during the incubation of *C. reinhardtii* strain cy6Nac2.49 (red) and it is parental wild-type (black) under the light/dark regime at 10 W·m^−2^ (**A**,**B**) 50 W·m^−2^.

Starch accumulation and acetate consumption were also measured under the same experimental conditions (Figure 4A,B). It should be noted these values are different from those seen in Figure 2, differences due to the fact that the results shown in Figure 4 were obtained at two different light intensities under a light:dark cycle whereas the results given in Figure 2 were obtained under a low light intensity (10 W·m^−2^) with continuous illumination. It is obvious that cultures incubated in the dark would consume acetate as well as some of the previously accumulated starch. Very little acetate was consumed by both the wild-type and cy6Nac2.49 cultures under low light conditions 10 W·m^−2^ (48 μmol·m^−2^·s^−1^), whereas after 95 h under the higher light intensity 50 W·m^−2^ (240 μmol·m^−2^·s^−1^), both cultures had consumed 70% of the initial acetate. Both the mutant and wild-type cultures accumulated higher amounts of biomass, estimated as cellular chlorophyll content, under 50 W·m^−2^ relative to 10 W·m^−2^ (Figure 4A,B). The wild-type showed greater growth than the cy6Nac2.49 strain under both conditions, producing 64% more biomass (on a chlorophyll basis) than the cy6Nac2.49 culture under low light conditions (10 W·m^−2^) and almost twice as much under the higher light condition (Figure 4A,B). Since the cultures were both diluted to the same initial chlorophyll concentration and final cell concentrations were not very high, chlorophyll serves as a good proxy for relative growth.

### 2.3. Differences in Initial Rates of Oxygen Evolution.

In order to measure any differences in the rates of respiration and photosynthesis between the wild-type and cy6Nac2.49 strain, short term experiments with a Clark-type O_2_ electrode were performed using two light intensities, 10 and 50 W·m^−2^ Indeed, significant differences were observed in the rates of photosynthesis between wild-type and cy6Nac2.49 cultures (Table 1). The rate of photosynthetic O_2_ evolution in the wild-type and cy6Nac2.49 cultures were measured in regular TAP medium by Clark-type O_2_ electrode upon illumination, and the respiration rate was determined in the dark period prior to illumination. Under both light intensities the rate of photosynthetic O_2_ evolution in the cy6Nac2.49 strain was more than 10 fold lower than in the wild-type, while the respiration rate in both cultures remained comparable (Table 1). The higher light intensity (50 W·m^−2^) promoted O_2_ evolution in the cy6Nac2.49 and wild-type cultures, and the rate of photosynthesis observed under 50 W·m^−2^ was about 4 times higher than that at 10 W·m^−2^ in both cy6Nac2.49 and wild-type cultures (Table 1). The low level of photosynthetic O_2_ evolution measured in the cy6Nac2.49 culture directly demonstrates that the introduced control of PSII activity prevents a rapid inhibition of hydrogen production by evolved oxygen. Thus, H_2_ evolution can be sustained for a longer period of time, as shown in the experiments under a light/dark regime (Figure 3A). 

## 3. Discussion

Green algae can grow under photoautotrophic, mixotrophic, photoheterotrophic and heterotrophic conditions [16,17]. Essential macro and micronutrient include sulfur, nitrogen, phosphorous, magnesium and trace elements like iron, copper, calcium, zinc, molybdenum, manganese and cobalt among others. In the absence of any of these nutrient, cell division is arrested and cultures stop growing [18]. In the case of sulfur deprivation, when washed free of sulfur, cultures stop growing within the first 5–20 h after transition into S-deprived medium [4,19]. Hydrogen production stops after 4–5 days of sulfur deprivation, and the cells have a spherical morphology with a significant reduction in cell mass [20]. Under phosphorous deprivation, cultures stop growing after 5 days, whereas H_2_ production stops after 10–12 days [5]. Nitrogen limitation causes cessation of cell growth after 2 days and H_2_ evolution stops after approximately 7 days [6]. In the case of magnesium deprivation, cultures stop growing after 7 days and H_2_ production lasts for an additional 7 days [7]. In all cases production of H_2_ eventually stops, despite the continuing presence of energy reserves in the form of starch, **triacyl glycerides** (TAGs), and acetate. This could be due to the toxic nature of the accumulated metabolites, or a result of the long-term consequences of nutrient deprivation [21]. The exact events leading to the termination of hydrogen photo-evolution are not entirely known.

These disadvantages suggest that it will be difficult to develop a practical process for H_2_ production in green algae using a nutrient depletion method. However, controllable expression of PSII could be used to reduce oxygen evolution to a rate below respiration, allowing cultures to naturally go anaerobic. The present work used cy6Nac2.49, a genetically modified strain of *C. reinhardtii* that activates photosynthesis in a cyclic manner in such a manner that photosynthesis is not active constitutively in the presence of light, but is turned on only in response to a metabolic trigger, anaerobiosis [11]. In this case the *nac2* gene, which stabilizes the mRNA of *psbD* encoding the reaction center polypeptide D2 of PSII, is regulated by an anaerobically induced promoter (Cyc6). Hence when oxygen is absent, expression of the *nac2* gene is enabled and photosynthesis is activated [11,12]. Once the oxygen level reaches the threshold for the Cyc6 promoter, transcription ceases and photosynthesis only occurs for a period of time until the PsbD protein is turned over. Once the produced oxygen is consumed by respiration, hydrogenase expression occurs and hydrogenase functions until oxygen reaches a low enough level for the *nac2* gene to be reactivated for another round of photosynthesis [11,12].

H_2_ production in regular TAP medium by the cy6Nac2.49 strain was examined under light/dark regime 10-h:14-h using two different light intensities, 10 and 50 W·m^−2^. The maximal rate of H_2_ production by the cy6Nac2.49 cultures was obtained at a light intensity of 10 W·m^−2^ and was equal to ~0.9 mmol·L^−1^, which is about 4.5 times higher than that obtained with wild-type cultures Figure 3A. However, the level of H_2_ in the cy6Nac2.49 strain at 50 W·m^−2^ was about ~0.3 mmol·L^−1^, slightly higher than what was produced by wild-type cultures Figure 3A. The high levels of H_2_ production in the cy6Nac2.49 strain indicate that it is able to sustain hydrogenase activity much longer than the wild-type, suggesting that driving *psbD* expression by an anaerobically induced promoter keeps oxygen production below the compensation point [11,12].

Previous work has demonstrated that the inhibitory effect of high light intensity on H_2_ photo-production is related to the enhanced O_2_ evolution activity of PSII, with the fast build-up of the O_2_ gas inactivating hydrogenase, stopping H_2_ production [22]. The optimal average light intensity for H_2_ production was shown to be 30–40 µE·m^−2^·s^−1^, which is equal to about ~10 W·m^−2^. Measurement of photosynthetic O_2_ evolution by the cy6Nac2.49 strain and wild-type with a Clark type electrode demonstrated a higher rate of O_2_ evolution in the cy6Nac2.49 strain at 50 W·m^−2^ compared to that at 10 W·m^−2^ (Table 1)*.* The level of photosynthetic O_2_ evolution of the cy6Nac2.49 strain was about 10 times lower than that of the wild-type, indicating an effective turn-off of PSII activity in the cy6Nac2.49 strain by the Cyc6 promoter under nutrient-replete conditions (Table 1)*.* Additionally, the low photosynthetic yield of cy6Nac2.49 cultures, measured as the maximum fluorescence, also suggests turn-off of PSII activity Figure 3B,C. As was mentioned above, maximum H_2_ production in cy6Nac2.49 cultures, ~0.9 mmol·L^−1^, was obtained in nutrient-replete conditions under a light/dark regime. For comparison, the hydrogen production of wild-type *C.reinhardtii* under *S*-deprivation is about ~3.27 mmol·L^−1^ [19]; under Mg-deprivation, ~6 mmol·L^−1^ [7]; under *P*-deprivation, ~2.45 mmol·L^−1^ [5]; under *N*-deprivation, ~1.5 mmol·L^−1^ [6]. Despite the lower level of H_2_ production demonstrated in the cy6Nac2.49 strain cultures under nutrient-replete conditions in comparison with the amounts of H_2_ obtained under nutrient-deprived conditions with wild-type cultures, application of controllable expression of PSII eliminates limitations associated with nutrient deprivation approaches. More importantly, under a light/dark regime H_2_ production by the cy6Nac2.49 cultures increased in every subsequent light period, and by the end of the last light period was 4.5-fold higher than the wild-type cultures. From this perspective, the application of the cy6Nac2.49 strain in long-term experiments for H_2_ production in fed-batch cultures is of future interest.

Further improvements of this system could come about by taking into account other factors besides the turn-off of PSII activity that could affect H_2_ production in *Chlamydomonas*. These could include considerations of starch accumulation, which serves as an additional source of electrons for hydrogen production, and the competition of hydrogen production with other electron sinks such as the Calvin–Benson cycle and cyclic electron flow. In principle genetic engineering could be applied to circumvent these limitations.

## 4. Materials and Methods

### 4.1. Cell Growth

Liquid cultures of *C. reinhardtii* strain cy6Nac2.49, kindly provided by Solarvest Bioenergy Inc., and its parental wild-type were cultivated photoheterotrophicaly, photomixotrophically, and autotrophicaly using, as appropriate, regular TAP and TP medium (Tris (2.42 g/L), phosphate solution (1 mL/L of K_2_HPO_4_ (288 g/L), KH_2_PO_4_ (144 g/L)), 1 mL/L of Hutner's trace elements). For autotrophic and photomixotrophic growth, the carbon dioxide concentration in the gas phase was adjusted, up to ~40%, at the beginning of the experiment by injecting pure carbon dioxide into the sealed 165 mL cylindrical vials which contained air. Vials were filled with 100 mL of culture and the pH was adjusted to 7.2, and the pressure in vials was equilibrated to atmospheric. As needed during cultivation, the pH was adjusted manually to 7.2. All cultures were shaken continuously on an orbital shaker (100 rpm) under continuous white light from LED lamps with an intensity of about 10 W·m^−2^ (approximately 48 μmol·m^−2^·s^−1^) or under a 10-h:14-h light/dark regime at two different light intensities, 10 and 50 W·m^−2^ (approximately 240 μmol·m^−2^·s^−1^). Growth was measured as an increase in cell density as monitored spectrophotometrically at 600 nm, a minimum in in vivo chlorophyll absorbance, and by measuring cellular chlorophyll (Chl) content. All of the experiments were carried out in triplicate and results are given as the mean + standard deviation. 

### 4.2. H_2_ Production under a Light/Dark Regime

Liquid cultures of *C. reinhardtii* strain cy6Nac2.49 and the parental wild-type, grown as described above with TAP medium, were inoculated by injecting the pre-inoculum into fresh TAP medium and dilution to a specific Chl concentration. Cultures were incubated in sealed 165 mL cylindrical vials under an air atmosphere at the start of the experiment, each vial contained 100 mL of culture. After an initial dark period (14 h), cultures were exposed to continuous white light (LED) for 10 h, followed again with the periods of dark (14 h) and light (10 h), with a total experimental duration of 100 h. Two light intensities, 10 W·m^−2^ (48 μmol m^−2^·s^−1^ and 50 W·m^−2^ (240 μmol·m^−2^·s^−1^, were used. The gas phase in the vials during the light period was analyzed by gas chromatography using a Shimadzu GC-8A (Shimadzu, Nakagyo-ku, Kyoto, Japan) equipped with 1 m × 0.3715 cm column packed with molecular sieve 5A using argon as carrier gas; the temperature of the injector and column were 110 °C and 60 °C, respectively and the current was 70 mA. Carbon dioxide production was measured in the same gas chromatograph equipped with 80/100 Porapak Q (Restek^TM^, Fishersci, Ottawa, ON, Canada) column using helium as a carrier gas, the temperature of injector and column were 100 °C and 40 °C, respectively, and the current was 100 mA.

During the light/dark periods, the quantum fluorescence yield of the cy6Nac2.49 and wild-type strains was measured as well as growth, estimated as total Chl. Photosynthetic yields were analyzed using a Handy PEA (Hansatech Instruments version 1.06 connected to a sensor of the same manufacture, King’s Lynn, Norfolk, UK) during the light/dark regime. The measurements were made through the glass at the bottom of the sealed bottles. A saturating pulse (1500 µmol photons m^−2^·s^−1^) of light (duration of 1 s) was applied to record Fv/Fm, the maximum fluorescence yield in the dark periods, while δFv/Fm’ represents the maximum fluorescence yield recorded during periods of illumination [23,24].

### 4.3. Oxygen Evolution and Consumption

Maximal O_2_ evolution in the light and respiration in the dark were measured with a Clark-type O_2_ electrode (Hansatech Instruments, King’s Lynn, Norfolk, UK). Measurements were conducted at 25 °C on vigorously stirred samples of the wild-type and cy6Nac2.49 strains in TAP medium. O_2_ evolution was determined when cultures were illuminated at light intensities of 10 and 50 W·m^−2^. Respiration rates were determined in the dark prior to measurements of photosynthetic oxygen evolution in illuminated cultures.

### 4.4. Other Analytical Procedures

The chlorophyll concentrations of 95% ethanol cell extracts were measured spectrophotometrically by the method of Spreitzer [25]. Samples were taken directly from vials with a sterile syringe and pelleted by centrifugation at 13000 rpm (MiniSpin, Eppendorf, NY, USA) for 3 min for starch, Chl and acetate measurements. The pellets and supernatants were separated and stored frozen at −20 °C until all samples were ready for processing. The amount of starch accumulated inside the cells was determined in the pellet according to the method developed by Gfeller and Gibbs (1984) [26], except that ethanol was used instead of methanol for cell disruption and pigment extraction. Acetate concentrations in the supernatants were determined using a colorimetric assay kit (BioVision Inc., Milpitas, CA, USA).

## 5. Conclusions

The high H_2_ production levels demonstrated by strain cy6Nac2.49 under a light/dark regime compared to what was observed with the wild-type suggests significant potential for its future application. In particular, with strain cy6Nac2.49, H_2_ production could be intimately coupled with the natural light/dark cycles, thus avoiding the use of the widely employed nutrient starvation approaches that require changes of growth media, consuming excess energy, leading to cell death, and which are difficult to apply in a cyclic manner. 

## Figures and Tables

**Table 1:** Changes in the rate of respiration and rate of photosynthesis in wild-type and cy6Nac2.49 strain of *C. reinhardtii* at different light intensities.

Light Intensity (W·m^−2^)	Rate of Respiration, µmol [O_2_ (mg·Chl)^−1^·h^−1^]		Rate of Photosynthesis, µmol [O_2_ (mg·Chl)^−1^·h^−1^]		Chl (a + b) [mg·(L^−1^)]	
	WT	Cy6Nac2.49	WT	Cy6Nac2.49	WT	Cy6Nac2.49
10 W·m^−2^	34.68 ± 0.12	28.44 ± 0.53	29.89 ± 0.82	2.10 ± 0.37	6.82 ± 0.52	6.68 ± 0.17
50 W·m^−2^	32.54 ± 0.87	28.37 ± 0.37	112.29 ± 0.73	9.31 ± 0.29	6.82 ± 0.52	6.68 ± 0.17

Data in the table are average of three independent experiments with standard deviations as defined in Microsoft Office Excel 2007.
